# Clinical outcomes of immune checkpoint inhibitors for patients with recurrent or metastatic head and neck cancer: real-world data in Korea

**DOI:** 10.1186/s12885-020-07214-4

**Published:** 2020-08-05

**Authors:** Hyera Kim, Minsuk Kwon, Binnari Kim, Hyun Ae Jung, Jong-Mu Sun, Se-Hoon Lee, Keunchil Park, Myung-Ju Ahn

**Affiliations:** 1 Division of Hematology-Oncology, Department of Medicine, Samsung Medical Center, Sungkyunkwan University School of Medicine, 81 Irwon-ro, Gangnam-gu, Seoul, 06351 Republic of Korea; 2 Division of Hematology-Oncology, Department of Internal Medicine, Keimyung University Dongsan Hospital, Daegu, Republic of Korea; 3 Department of Pathology and Translational Genomics, Samsung Medical Center, Sungkyunkwan University School of Medicine, Seoul, Republic of Korea; 4 Center of Companion Diagnostics, Samsung Medical Center, Seoul, Republic of Korea

**Keywords:** Immune checkpoint inhibitor, Head and neck cancer, Pembrolizumab, Nivolumab

## Abstract

**Background:**

Anti-PD1 inhibitors have been approved for the treatment of recurrent or metastatic head and neck cancer (HNC), as a result of Global Phase III trials. However, the clinical outcomes of immune checkpoint inhibitors in patients who are not eligible for clinical trials or have various medical conditions have not been fully elucidated.

**Methods:**

We retrospectively reviewed 46 patients with recurrent or metastatic HNC who received pembrolizumab or nivolumab between June 2016 and June 2019.

**Results:**

Thirty-five patients had head and neck squamous cell carcinoma (HNSCC) affecting the oropharynx, oral cavity, hypopharynx, larynx, nasal cavity, or paranasal sinuses, and eleven patients had nasopharyngeal cancer (NPC). The median progression-free survival (PFS) and overall survival (OS) were 3.7 months and 6.8 months, respectively, for patients with HNSCC, and 4.3 months and 11.8 months, respectively, for patients with NPC. The objective response rate (ORR) in all patients was 21%. Of 30 patients with HNSCC, 5 patients achieved complete response and 2 achieved partial response (ORR 23%); 1 of 8 NPC patients achieved partial response (13%). Patients who previously underwent radiotherapy had better OS than those who did not (median OS, 7.6 months vs. 2.3 months, *p* = 0.006). OS was longer in patients treated with pembrolizumab than in those treated with nivolumab (median OS, 11.8 months vs. 6.8 months, *p* = 0.017).

**Conclusion:**

Consistent with previous reports, immune checkpoint inhibitors showed promising efficacy in patients with previously treated recurrent or metastatic HNC in a real-world setting.

## Background

Head and neck cancer (HNC) occurs in complex sites of the head and neck and has various types of histology, which consist mainly of squamous cell carcinoma (SCC). Over 50% of patients with HNC present with locally advanced stage, and half experience relapse within 3 years [1, 2]. The EXTREME regimen, including fluorouracil, platinum, and cetuximab, resulted in a 2.7-month improvement of overall survival compared with the regimen, including fluorouracil and platinum, leading to its approval as a first-line chemotherapy for recurrent or metastatic head and neck cancer (RMHNC) [3]. However, during the last decade, there has been little improvement in second-line therapies with low response rates and high toxicity for RMHNC [4, 5]. Thus, after disease progression on a platinum-based regimen, there are very limited treatment options for RMHNC, and the prognosis is poor, with a median overall survival (OS) of less than 7 months [6–8].

Immune checkpoint inhibitors have become a standard therapy for various types of cancer [9–11]. The improved outcomes of anti-PD1 inhibitors in head and neck squamous cell carcinoma (HNSCC) were demonstrated by two landmark randomized Phase 3 trials. Pembrolizumab and nivolumab had favorable safety profiles and produced clinically meaningful improvements in OS (pembrolizumab, hazard ratio [HR] 0.80, 95% confidence interval [CI] 0.65–0.98, *p* = 0.0161; nivolumab, HR 0.70, 97.73% CI 0.51–0.96, *p* = 0.01) compared with investigator’s choice in patients with recurrent or metastatic head and neck squamous cell carcinoma (RMHNSCC) in the Phase 3 trials KEYNOTE-040 [12] and CheckMate-141 [13], respectively.

In general, prospective Phase 3 trials in HNSCC require strict eligibility criteria, such as good performance status, predefined disease site, or limited lines of previous therapy. Thus, patients with non-SCC and other subtypes, such as cancer of the nasal cavity/paranasal sinuses and nasopharynx, are usually excluded. Therefore, the “real-world” clinical outcomes of immune checkpoint inhibitors in patients who are not eligible for clinical trials are very limited.

Here, we have evaluated the efficacy of pembrolizumab or nivolumab in patients with RMHNC in a real-world setting.

## Methods

We retrospectively analyzed 46 patients with RMHNC treated at Samsung Medical Center who received pembrolizumab or nivolumab from June 2016 to June 2019. Patients had pathologically confirmed the head and neck cancer, except for salivary gland cancer, and had experienced relapse or disease progression after or during previous treatment, including chemotherapy, radiotherapy, and chemoradiotherapy. There was no limitation to the number of lines of chemotherapy. Patients received 200 mg pembrolizumab every 3 weeks or 3 mg/kg nivolumab every 2 weeks.

Medical records were reviewed for the following characteristics: age; sex; smoking history; Eastern Cooperative Oncology Group Performance Status (ECOG PS) prior to the use of immune checkpoint inhibitors; the date of diagnosis, last follow-up visit, or death; primary tumor location; histology; status of human papillomavirus (HPV) and Epstein–Barr virus (EBV); PD-L1 expression; and prior treatment. HPV expression was assessed using p16 immunohistochemistry or real-time polymerase chain reaction (PCR), and EBV expression was assessed using in situ hybridization. PD-L1 expression was tested using the PD-L1 IHC 22C3 pharmDx assay and most patients were classified by the combined positive score (CPS), defined as the number of PD-L1-positive cells (tumor cells, lymphocytes, and macrophages) divided by the total number of viable tumor cells presented by percentage.

Treatment response was assessed by CT scans in accordance with Response Evaluation Criteria in Solid Tumors (RECIST version 1.1). The objective response rate (ORR) was defined as the percentage of patients with a complete or partial response (CR or PR, respectively). The intervals between the time from the first cycle of immunotherapy and that of death alone [overall survival (OS)], disease progression, or death [progression-free survival (PFS)] were calculated for each patient. The duration of response (DOR) was defined as the time from first CR or PR to progressive disease (PD) or death. Patients discontinued treatment owing to disease progression, intolerable toxicity, or poor general condition. The data was collected until 28 October 2019. This study was approved by the Institutional Review Board of Samsung Medical Center; the requirement for written informed consent was waived owing to the retrospective nature of the study.

All statistical analyses were computed using IBM SPSS Statistics for Windows, version 25.0 (IBM Corp., Armonk, NY, USA). PFS, OS, and DOR were calculated using the Kaplan-Meier method. A Cox proportional hazards regression model was used in univariate analyses. The results were presented as HRs and 95% CIs. The significant differences were assigned at *P* values of less than 0.05.

## Results

### Patient characteristics

In total, 46 patients with RMHNC who received pembrolizumab or nivolumab were included in this study; the characteristics of patients are presented in Table 1. Of the 46 patients, 35 had HNSCC, and 11 had nasopharyngeal cancer (NPC); 8 (72.7%) had non-keratinizing carcinoma and 3 (27.3) had other histologies (poorly differentiated carcinoma [*n* = 1], large cell neuroendocrine carcinoma [*n* = 1], and adenoid cystic carcinoma [*n* = 1]). The median age at immunotherapy was 57.8 years (range, 39–73 years of age) for patients with HNSCC and 47.4 years (range, 16–74 years of age) for patients with NPC. Most patients were men (HNSCC, 71.4%; NPC, 81.8%). Patients with HNSCC (62.9%) and NPC (54.6%) had a smoking history. Eight patients (22.9%) with HNSCC had an ECOG PS score of 2. In HNSCC, the primary tumor locations included hypopharynx/larynx (*n* = 5), oropharynx/oral cavity (*n* = 15), nasal cavity/paranasal sinuses (*n* = 12), and others (*n* = 3). Twelve patients (80.0%) with oropharyngeal and oral cavity cancer were examined for HPV expression, and 5 (33.3%) had HPV-associated disease. Nine patients (81.8%) with NPC were examined for EBV expression; 8 (88.9%) patients were positive. In addition, PD-L1 expression was examined in 15 (42.9%) patients with HNSCC and 8 (72.7%) patients with NPC, respectively. A PD-L1 CPS of 1 or higher was detected in 12 (80.0%) patients with HNSCC and 6 (75.0%) patients with NPC; 11 patients had a PD-L1 CPS of 20 or higher.

**Table 1:** Characteristics of patients with immune checkpoint inhibitors in head and neck cancer

	HNSCC (***n*** = 35)		NPC (***n*** = 11)	
**Age (years)**	57.8 (39–73)		47.4 (16–74)	
**Sex**				
**Male**	25 (71.4)		9 (81.8)	
**Female**	10 (28.6)		2 (18.2)	
**Smoking**				
**Current**	7 (20.0)		3 (27.3)	
**Former**	15 (42.9)		3 (27.3)	
**Never**	13 (37.1)		5 (45.5)	
**ECOG**				
**1**	27 (77.1)		11 (100.0)	
**2**	8 (22.9)		0 (0.0)	
**Primary tumor location**	**Hypopharynx/Larynx**	5	**Nasopharynx**	11
	**Oropharynx/Oral cavity**	15		
	**Nasal cavity/Paranasal sinuses**	12		
	**Others**	3		
**Histology**	**SQ**	32 (91.4)	**Non-keratinizing carcinoma**	8 (72.7)
	**Others**	3 (8.6)	**Others**	3 (27.3)
	**HPV (oropharynx/oral cavity)**		**EBV**	
**Positive**	5 (33.3)		8 (72.7)	
**Negative**	7 (46.7)		1 (9.1)	
**NA**	3 (20.0)		2 (18.2)	
**PD-L1 22C3 (CPS)**				
**< 1**	3		2	
**≥ 1**	12 (≥ 20:8)		6 (≥ 20:3)	
**NA**	20		3	
**Prior treatment**				
**Surgery → CCRT/RT**	14 (40.0)		0	
**CCRT/RT → Surgery**	4 (11.4)		0	
**CCRT**	10 (28.6)		6 (54.5)	
**RT**	5 (14.3)		1 (9.1)	
**Induction chemotherapy**	9 (25.7)		1 (9.1)	
**Adjuvant chemotherapy**	1 (2.9)		2 (18.2)	
**Platinum-refractory**				
**Yes**	29 (82.9)		10 (90.9)	
**No**	5 (14.3)		1 (9.1)	
**No exposure to platinum**	1 (2.9)		0 (0.0)	
**Prior palliative chemotherapy lines**	1 (0–4)		2 (1–4)	
**Prior Cetuximab + Platinum**	13 (37.1)		0 (0.0)	
**Immunotherapy**				
**Nivolumab**	29 (82.9)		3 (27.3)	
**Pembrolizumab**	6 (17.1)		8 (72.7)	
**No. of cycles**	3 (1–19)		3 (1–24)	

*Abbreviations*: *HNSCC* head and neck squamous cell carcinoma, *NPC* nasopharyngeal cancer, *ECOG* Eastern Cooperative Oncology Group, *SQ* squamous cell carcinoma, *HPV* human papillomavirus, *EBV* Epstein–Barr virus, *NA* not available, *CPS* combined positive score, *CCRT* concurrent chemoradiotherapy

Surgery of the primary tumor was performed in 18 (51.4%) patients with HNSCC, and concurrent chemoradiotherapy or radiotherapy was performed in 33 (94.3%) patients with HNSCC and 7 (63.6%) patients with NPC. Among 14 patients who received concurrent chemoradiotherapy or radiotherapy after surgery, 10 patients had remnant or recurrent tumor after surgery. Chemotherapy with cetuximab and platinum before immunotherapy was administered in 13 (37.1%) patients with HNSCC and no patients with NPC. Six patients received immunotherapy as the first systemic therapy, and all these patients were in the HNSCC group. The median number of lines of prior palliative chemotherapy and the median number of cycles of immunotherapy were 1 (0–4) and 3 (1–19) for patients with HNSCC and 2 (1–4) and 3 (1–24) for patients with NPC, respectively. Twenty-nine patients (82.9%) with HNSCC and 3 (27.3%) patients with NPC had received nivolumab, and others had received pembrolizumab. In HNC, 39 (84.8%) patients were platinum-refractory and 7 (15.2%) patients were not platinum-refractory.

The median follow-up duration from the start date of immunotherapy for all patients was 4.8 months (range, 0.5–19.8 months) and 3.8 months (range, 0.4–18.4 months) for the monitoring of OS and PFS, respectively. At the time of cut-off, death had occurred in 30 (65.2%) of 46 patients, and 43 (93.5%) patients had discontinued the immunotherapy, mostly as a result of progressive disease (*n* = 26).

### Efficacy of immune checkpoint inhibitors in patients with head and neck cancer

**Fig. 1:**
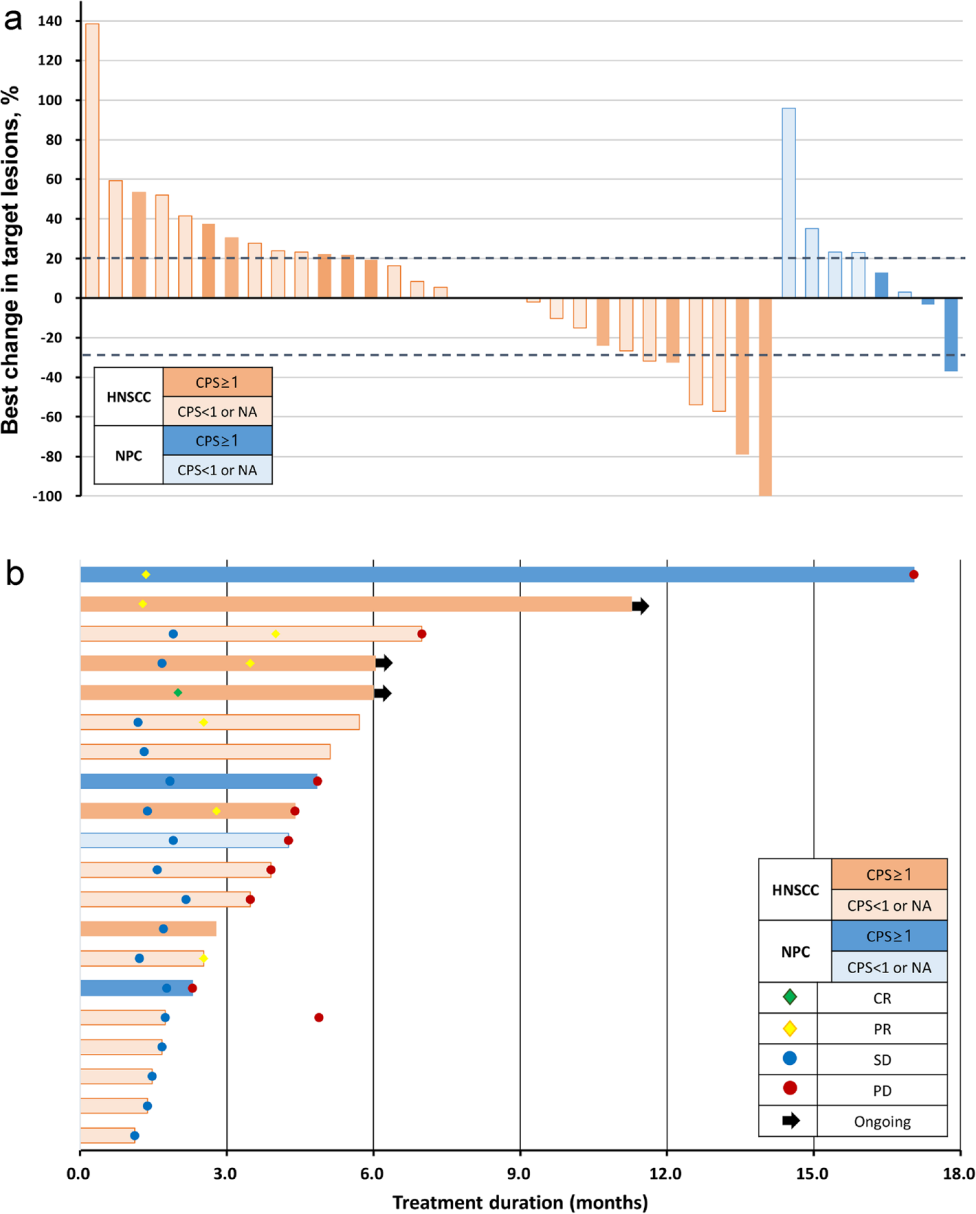
The efficacy of immune checkpoint inhibitors in patients with head and neck cancer. **a** The best percentage change from baseline in target lesion size was assessed for patients with at least one follow-up scan of the target lesions (*n* = 38). **b** Treatment exposure and response duration for patients with at least stable disease as per RECIST v1.1 (*n* = 20). *HNSCC* head and neck squamous cell carcinoma, *NPC* nasopharyngeal cancer, *CPS* combined positive score, *NA* not available, *CR* complete response, *PR* partial response, *SD* stable disease, *PD* progressive disease

The ORR and DOR in all patients were 21% and 4.4 months (range 1.6–15.7 months), respectively; 13 and 8% of patients achieved CR and PR, respectively. In addition, 37 and 42% of patients had SD and PD, respectively, as the best response. Response evaluations were available for 82.6% of patients (Table 2). Of the 30 patients with HNSCC, 5 patients (17%) achieved complete response, and 2 patients (7%) achieved partial response, with an ORR of 23% and a DOR of 4.4 months, whereas 1 patient (13%) of 8 with NPC achieved partial response, and the DOR was 15.7 months. Decrease in the size of target lesion from baseline was observed in 13 patients (34.2%) among all patients with available data, including 36.7% of patients with HNSCC and 25.0% of those with NPC (Fig. 1a). Among the 20 responders (including those with CR, PR, and SD), the median time to best response was 1.8 months (range, 1.1–4.0 months) (Fig. 1b). Among the responders, 3 patients remained on immunotherapy, including 2 patients with PR and 1 patient with CR.

**Table 2:** The response rate of patients with immune checkpoint inhibitors in head and neck cancer

Histology	Response, n/N (%)					Median DOR, months
	ORR	CR	PR	SD	PD	
**All patients (*n*** **= 38)**	8/38 (21)	5/38 (13)	3/38 (8)	14/38 (37)	16/38 (42)	4.4
**HNSCC (*n*** **= 30)**	7/30 (23)	5/30 (17)	2/30 (7)	11/30 (37)	12/30 (40)	4.4
**Hypopharynx/Larynx**	0/4 (0)	0/4 (0)	0/4 (0)	1/4 (25)	3/4 (75)	–
**Oropharynx/Oral cavity**	5/13 (38)	0/13 (0)	5/13 (38)	4/13 (31)	4/13 (31)	4.4
**Nasal cavity/Paranasal sinuses**	2/11 (18)	1/11 (9)	1/11 (9)	5/11 (45)	4/11 (36)	3.0
**Others**	0/2 (0)	0/2 (0)	0/2 (0)	1/2 (50)	1/2 (50)	–
**Nasopharyngeal cancer (*n*** **= 8)**	1/8 (13)	0/8 (0)	1/8 (13)	3/8 (38)	4/8 (50)	15.7

*Abbreviations*: *ORR* objective response rate, *CR* complete response, *PR* partial response, *SD* stable disease, *PD* progressive disease, *DOR* duration of response, *HNSCC* head and neck squamous cell carcinoma

**Fig. 2:**
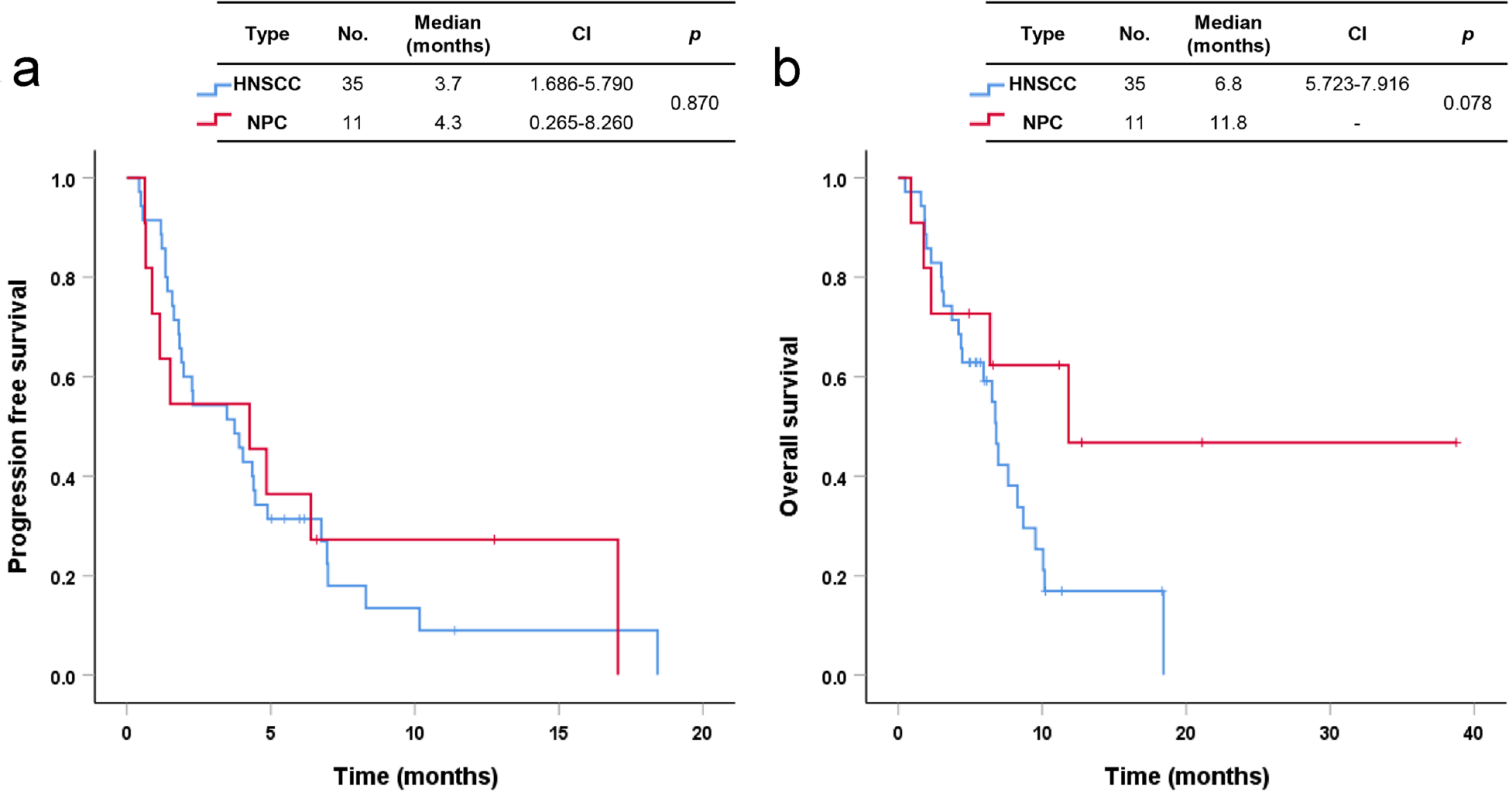
Progression-free survival (**a**) and overall survival (**b**) in patients with head and neck cancer treated with immune checkpoint inhibitors. *HNSCC* head and neck squamous cell carcinoma, *NPC* nasopharyngeal cancer

**Fig. 3:**
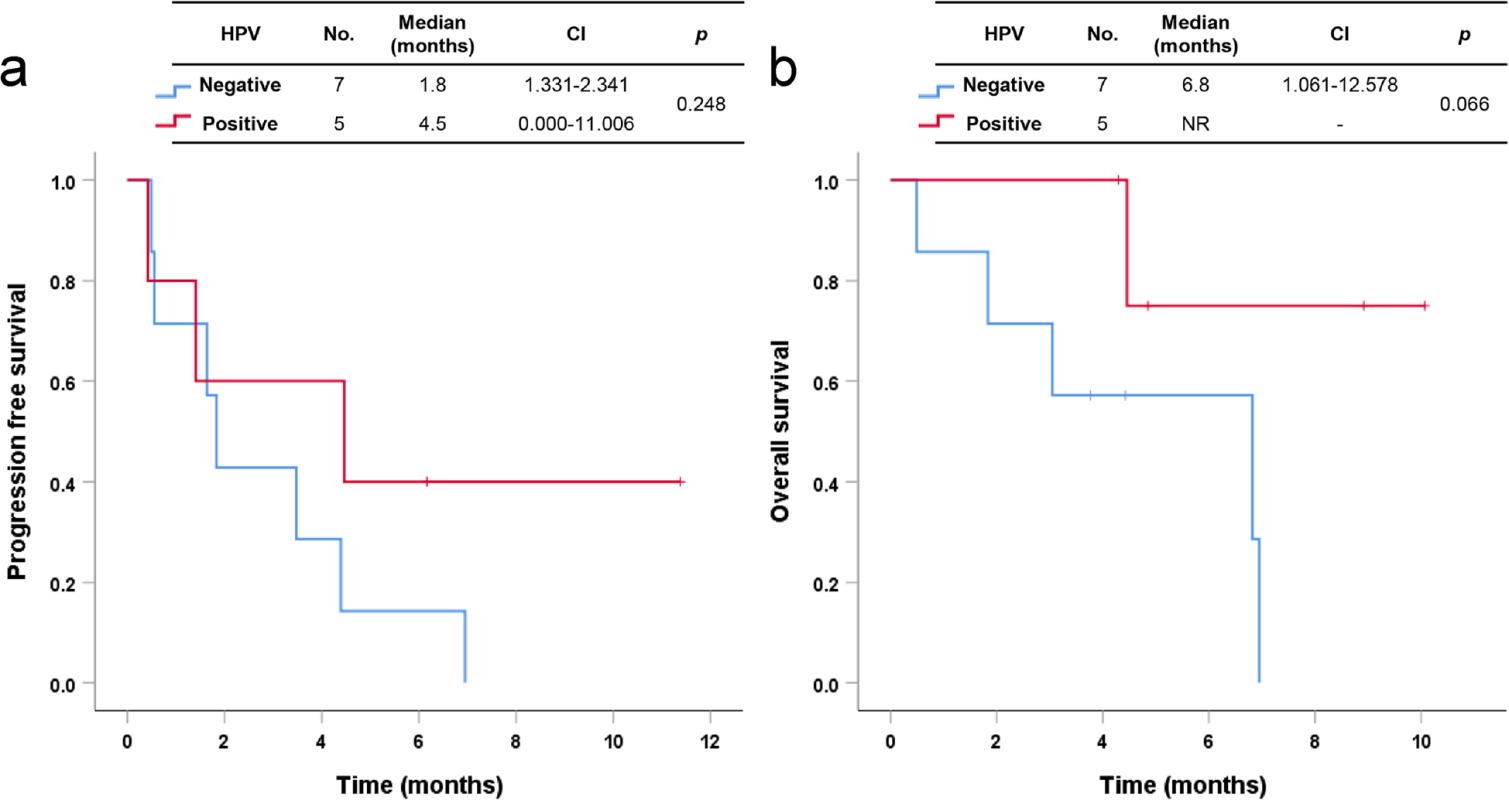
Progression-free survival (**a**) and overall survival (**b**) in patients with cancers of the oral cavity and oropharynx treated with immune checkpoint inhibitors according to the HPV expression. *HPV* human papillomavirus

**Fig. 4:**
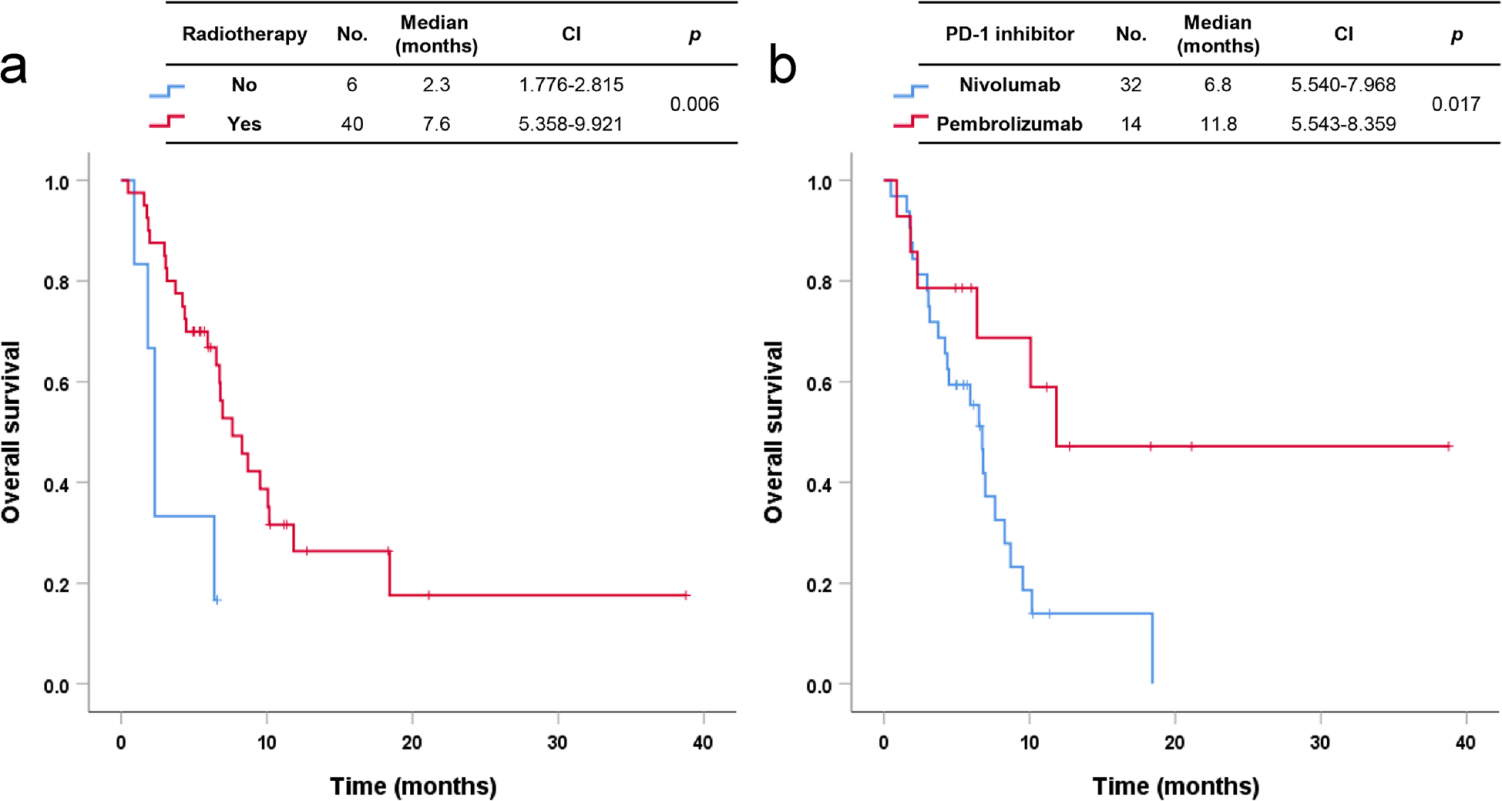
Overall survival by immune checkpoint inhibitors according to previous radiotherapy treatment (**a**) and type of PD-1 inhibitor (**b**) in patients with head and neck cancer

The median PFS and OS of patients with HNSCC were 3.7 months (95% CI 1.686–5.790) and 6.8 months (95% CI 5.723–7.916), respectively. The median PFS and OS of patients with NPC were 4.3 months (95% CI 0.265–8.260) and 11.8 months, respectively (Fig. 2a, b). In cancers of the oropharynx and oral cavity, the median PFS and OS of patients with HPV-associated disease were 4.5 months (95% CI 0.000–11.006) and not reached, respectively. Patients with HPV-associated disease tended to have better OS and PFS than patients with non-HPV-associated disease, but there was no statistical significance in results (Fig. 3a, b). Using univariate analysis, we found that three prognostic factors were associated with OS: ECOG (≥2, HR 2.724, CI 1.195–6.208, *p* = 0.017), history of radiotherapy (Yes, HR 0.262, CI 0.093–0.736, *p* = 0.011), and type of PD-1 inhibitor (pembrolizumab, HR 0.336, CI 0.132–0.852, *p* = 0.022). The radiotherapy group had better OS than the group without a history of radiotherapy (median OS, 7.6 months vs. 2.3 months, *p* = 0.006) (Fig. 4a). In addition, the pembrolizumab group had better OS than the nivolumab group (median OS, 11.8 months vs. 6.8 months, *p* = 0.017) (Fig. 4b). There were not significant differences in survival outcomes between platinum-refractory carcinoma and non-platinum-refractory carcinoma patients (median PFS, 3.5 months vs. 10.2 months, *p* = 0.085; median OS, 6.8 months vs. 10.2 months, *p* = 0.306).

## Discussions

In the present study, we revealed that patients with RMHNSCC receiving pembrolizumab or nivolumab exhibited a PFS of 3.7 months, an OS of 6.8 months, and an ORR of 23% for patients in a real-world setting. Further, the median PFS was 4.3 months and the median OS was 11.8 months in patients with NPC. In clinical practice, we often encounter patients with RMHNC who do not meet the eligibility criteria for clinical trials, such as the KEYNOTE-040 and CheckMate-141 trials. Our study included patients with carcinoma of the nasal cavity/paranasal sinuses, nasopharynx, external auditory canal, and other rare sites; histology of non- SCC; and non-platinum-refractory carcinoma. Recently, a few retrospective studies were investigated to evaluate the efficacy of nivolumab in RMHNC. Hori et al. [14] reviewed 93 patients with RMHNC, including non-SCC and patients not exposed to platinum, and reported an ORR of 18% and a PFS of 4.3 months for all patients. In addition, 100 patients with RMHNC, including those with non-SCC and cancer of the nasopharynx, were analyzed by Okamoto [15], who reported an ORR, median PFS, and OS of 13.5%, 3.7 months, and 9.6 months, respectively, which were in line with the results of our study conducted in a real-world setting.

In HNC, approximately one-quarter of cases are related to human papillomavirus (HPV) infection, which is predominantly found in the oropharynx and oral cavity and is associated with favorable prognosis [16]. However, it is still controversial as to whether HPV status should be considered for the use of immune checkpoint inhibitors. In KEYNOTE-012 [17], response rates were higher in patients with HPV-associated cancer compared with patients with non-HPV associated cancer, with ORR of 24% (95% CI, 13–40%) and 16% (95% CI, 10–23%), respectively. In contrast, in the CheckMate-141 trial, patients received a consistent benefit from nivolumab, regardless of HPV status (HPV-negative patients, HR 0.59, 95% CI: 0.38–0.92; HPV-positive patients, HR 0.60, 95% CI: 0.37–0.97) [18]. In our study, there was a trend toward favorable PFS or OS in HPV-associated disease than non-HPV-associated disease, but the trend was not significant. Therefore, HPV status should not limit the use of immune checkpoint inhibitors, as patients with both HPV-positive and HPV-negative RMHNC may experience survival benefit with the available PD-1 inhibitors.

Of note, this study demonstrated the importance of previous radiotherapy associated with a favorable clinical outcome for immune checkpoint inhibitors. Patients with a history of concurrent chemoradiotherapy or radiotherapy alone had a longer OS with immunotherapy. Hori et al. [14] previously reported that a history of radiotherapy for the primary tumor was associated with a better PFS (HR 1.95, 95% CI 1.07–3.55, *p* = 0.028) in 93 RMHNC patients who received nivolumab. A possible explanation for this is that radiotherapy at the tumor site could enhance the presentation of tumor cell-derived antigens, giving rise to primed cytotoxic T cells and leading to local and systemic effects on both local and metastatic disease via the abscopal effect [19]. Immunotherapy, such as blockade of the PD-1/PD-L1 signaling pathway, may provide an opportunity to boost the abscopal effect [20]. Twyman-Saint Victor et al. [21] demonstrated that radiotherapy, in combination with inhibitors of cytotoxic T lymphocyte-associated antigen (CTLA4) and PD-L1, enhanced immunity through distinct mechanisms and increased abscopal response rates in melanoma. Our results could provide indirect evidence of this effect in RMHNC.

The PD-1 inhibitors, nivolumab and pembrolizumab, each resulted in similar OS in Phase 3 trials: 7.5 months (95% CI 5.5–9.1) for nivolumab and 8.4 months (95% CI 6.4–9.4) for pembrolizumab, respectively [12, 13]. Interestingly, our study revealed that the two treatment groups of PD-1 inhibitors had a significant difference in OS (nivolumab vs. pembrolizumab, median OS, 6.8 months vs. 11.8 months, *p* = 0.017). This result should be interpreted with caution because of several confounding factors, such as imbalanced distribution of HNSCC and NPC and difference in patient population.

There were several limitations to this study. First, we divided patients with HNC into those with HNSCC and those with NPC and compared parameters between these groups, but the number of patients was too small to make reliable comparisons between the two groups. Second, we failed to define the interaction between the efficacy of immunotherapy and PD-L1 expression owing to limited sample size and insufficient tissue samples. Given that several patients with a PD-L1 CPS score of 1 or higher achieved a deep response, further studies are needed to solidify the importance of PD-L1 expression for the efficacy of immunotherapy in patients with RMHNC. Finally, the effects of immune checkpoint inhibitors may have been underestimated because this study included heavily treated patients with a maximum of four lines of prior palliative chemotherapy.

## Conclusion

Immune checkpoint inhibitors, pembrolizumab or nivolumab, showed promising efficacy in patients with RMHNC in a real-world setting, and these findings are consistent with those reported previously.

## Data Availability

The datasets used and analyzed in the current study are available from the corresponding author on reasonable request.

## Abbreviations

HNC: Head and neck cancer
HNSCC: Head and neck squamous cell carcinoma
NPC: Nasopharyngeal cancer
PFS: Progression-free survival
OS: Overall survival
ORR: Objective response rate
HR: Hazard ratio
CI: Confidence interval
RMHNC: Recurrent or metastatic head and neck cancer
RMHNSCC: Recurrent or metastatic head and neck squamous cell carcinoma
ECOG PS: Eastern Cooperative Oncology Group Performance Status
HPV: Human papillomavirus
EBV: Epstein–Barr virus
PCR: Polymerase chain reaction
CPS: Combined positive score
CR: Complete response
PR: Partial response
DOR: Duration of response
PD: Progressive disease
